# Comparison Between Two Pharmacologic Strategies to Alleviate Rewarming Shock: Vasodilation vs. Inodilation

**DOI:** 10.3389/fmed.2020.566388

**Published:** 2020-11-12

**Authors:** Brage Håheim, Timofei Kondratiev, Erik Sveberg Dietrichs, Torkjel Tveita

**Affiliations:** ^1^Anesthesia and Critical Care Research Group, Department of Clinical Medicine, UiT, The Arctic University of Norway, Tromsø, Norway; ^2^Experimental and Clinical Pharmacology Research Group, Department of Medical Biology, UiT, The Arctic University of Norway, Tromsø, Norway; ^3^Division of Surgical Medicine and Intensive Care, University Hospital of North Norway, Tromsø, Norway

**Keywords:** hypothermia, nitroprusside, levosimendan, rewarming shock, targeted therapeutic strategies, microcirculation

## Abstract

Rewarming from hypothermia is often challenged by coexisting cardiac dysfunction, depressed organ blood flow (OBF), and increased systemic vascular resistance. Previous research shows cardiovascular inotropic support and vasodilation during rewarming to elevate cardiac output (CO). The present study aims to compare the effects of inodilatation by levosimendan (LS) and vasodilation by nitroprusside (SNP) on OBF and global oxygen transport during rewarming from hypothermia. We used an *in vivo* experimental rat model of 4 h 15°C hypothermia and rewarming. A stable isotope-labeled microsphere technique was used to determine OBF. Cardiac and arterial pressures were monitored with fluid-filled pressure catheters, and CO was measured by thermodilution. Two groups were treated with either LS (*n* = 7) or SNP (*n* = 7) during the last hour of hypothermia and throughout rewarming. Two groups served as hypothermic (*n* = 7) and normothermic (*n* = 6) controls. All hypothermia groups had significantly reduced CO, oxygen delivery, and OBF after rewarming compared to their baseline values. After rewarming, LS had elevated CO significantly more than SNP (66.57 ± 5.6/+30% vs. 54.48 ± 5.2/+14%) compared to the control group (47.22 ± 3.9), but their ability to cause elevation of brain blood flow (BBF) was the same (0.554 ± 0.180/+81 vs. 0.535 ± 0.208/+75%) compared to the control group (0.305 ± 0.101). We interpret the vasodilator properties of LS and SNP to be the primary source to increase organ blood flow, superior to the increase in CO.

## Introduction

Clinical presentation of accidental hypothermia and rewarming is associated with hypotension, hypoperfusion, and vital organ injury (1, 2). Coined *rewarming shock*, this unstable hemodynamic state (3–5) contributes to the lethality of 28–35% in accidental hypothermia patients (6, 7). The underlying mechanisms of rewarming shock are yet not fully understood. Clinical experience and experimental studies have identified hypothermia-induced cardiac dysfunction and elevated systemic vascular resistance (SVR) as fundamental mechanisms (5, 8). Hypothermia-induced cardiac dysfunction has been linked to inotropic failure and dysregulation of myocardial beta-receptors. The current European guidelines for resuscitation during hypothermia focus on mechanical circulatory support in unstable patients and restrictive adrenergic intervention, significantly below 30°C (9). Thus, experimental studies are needed to improve our understanding of rewarming shock and identify possible pharmacological treatment options.

A combination of positive inotropy and vasodilation, i.e., inodilation, with levosimendan (LS), or vasodilation only with sodium nitroprusside (SNP), have both demonstrated to elevate cardiac output (CO) and reduce SVR in experimental models of hypothermia and rewarming (8, 10, 11). The results show LS and SNP to elevate CO by +166 and +77%, respectively (8, 10). LS increases Ca^2+^ sensitivity of the cardiac contractile apparatus and open ATP-dependent K^+^-channels in smooth muscle, resulting in elevated cardiac inotropy and vasodilation. LS also inhibits phosphodiesterase-3, elevating cyclic adenosine monophosphate (cAMP), promoting additional positive inotropy and vasodilation (12). SNP vasodilates by activating smooth muscle cyclic guanosine monophosphate (cGMP) (13), and *in vivo* studies show no changes in cardiac contractility (14).

Organ blood flow (OBF) and oxygen transport (DO_2_) are essential factors in the treatment of critical care patients (15). The pharmacological elevation of OBF improves short-term organ function and reduce mortality of patients in circulatory shock (16, 17). While studies conducted during normothermic conditions have shown elevated OBF by both LS and SNP (18–20), little knowledge exists on their effects during rewarming from hypothermia. It is therefore vital to assess if their beneficial effects on global hemodynamic function translate into improved OBF (8, 10). With this in mind, we hypothesize that the combined inotropic and vasodilatory effects of LS improve OBF more than isolated vasodilation by SNP after rewarming from hypothermia. To test our hypothesis, we used an *in vivo* rat model instrumented for measurements of hemodynamic function and OBF during cooling, 3 h stable hypothermia, and rewarming. Pharmacologic interventions were instituted 1 h before rewarming and continued throughout the rewarming process.

## Materials and Methods

### Experimental Design

The main aim of this study was to investigate if the beneficial effects of LS and SNP on cardiac function translate into improved organ perfusion during hypothermia and rewarming. Four experimental groups were included: control (*n* = 7), Levosimendan (*n* = 7), Nitroprusside (*n* = 7), and Normothermic control (*n* = 6).

### Control (*n* = 7)

The animals were cooled to and kept at 15°C for 3 h. After 2 h, the animals received a bolus dose of 0.33 ml of 5% glucose over 10 min, followed by a continuous infusion of 0.5 ml/h during the last hour of hypothermia and until rewarming was completed at 37°C.

### Levosimendan (*n* = 7)

The animals were cooled to and kept at 15°C for 3 h. After 2 h, the animals received a bolus dose of 24 μg/kg/min levosimendan over 10 min, followed by a continuous 0.6 μg/kg/min infusion during the last hour of hypothermia and throughout rewarming (10).

### Nitroprusside (*n* = 7)

The animals were cooled to and kept at 15°C for 3 h. After 2 h, an infusion of nitroprusside was started at 0.625 μg/kg/min. During rewarming, the dose was titrated to reduce mean arterial pressure (MAP) by 30% compared to historical controls (8). On average, each rat received 0.178 mg of nitroprusside.

### Normothermic Control (*n* = 6)

The animals were kept at 37°C for 5 h. After 2.5 h, the animals received a 10-min bolus infusion of 2.0 ml/h glucose (5%), followed by a 0.5 ml/h infusion lasting throughout the remaining experiment.

### Hemodynamic Data

Arterial pressure was obtained with a fluid-filled 22G cannula inserted in the left femoral artery and connected to a fluid manometer. Left ventricular pressure was obtained with a fluid-filled catheter inserted into the right carotid artery and advanced to the left ventricle under pressure guidance and connected to a manometer.

CO was measured with the thermodilution method, by injecting 0.15 ml of precooled (5°C) 0.9% saline through the jugular vein (21). The rapid change in temperature was recorded by a thermocouple, inserted into the left femoral artery, and advanced to the ascending aortic arch. Thermodilution curves were recorded and analyzed in LabChart 8.0. In the hypothermia groups, CO was measured at 37, 30, 22, and 15°C during cooling and rewarming. In the normothermia group, CO was measured at baseline (37°C_BL_) and, after that, hourly till final recording (37°C_5h_). Heart rate (HR) was calculated based on the femoral pressure signal, and SVR, stroke volume (SV), and cardiac index (CI) were calculated using the following formulas: SVR = MAP/CO, SV = CO/HR, and CI = CO/9.83^*^[body weight^(2/3)^].

To investigate organ blood flow, we applied the stable isotope microsphere technique (22). A volume of 0.5 ml, containing 250,000 microspheres/ml (BioPal Mi, USA), labeled with either lutetium or samarium, was injected into the left ventricle using the catheter. Simultaneously, a reference sample, 0.5 ml/min, was drawn from the left femoral artery (2, 23, 24). After euthanasia, the brain and cerebellum, heart, kidneys, liver, and stomach were harvested, washed in SanSaline (BioPal), weighed, and dried. Quantification of microspheres in tissue and blood samples was done by BioPal (Mi, USA). Later, OBF was calculated by normalizing organ microsphere concentration (disintegration per minute/g) with the microsphere concentration of the reference sample (disintegration per minute/ml/min) (22). In the hypothermia groups, OBF was measured during rewarming at 30 and 37°C. In the normothermia group (37°C), OBF was measured at baseline.

### Biochemical Data

Arterial blood was analyzed for pO_2_, pCO_2_, oxygen saturation (SaO_2_), pH, hemoglobin (Hb), hematocrit, and lactate. ${\text{HCO}}_{3}^{-}$ and base excess were automatically calculated based on the measured data (Rapidlab 800, Chiron Diagnostics). Blood gases were analyzed at 37°C and not corrected for core temperature (25). In the hypothermia groups, arterial blood gas analyses were obtained at baseline, during cooling at the start of stable hypothermia (15°C_0_), during rewarming at 30°C (30°C_RW_), and after rewarming (37°C_RW_). At 37°C_RW_, a venous blood gas was also sampled. In the normothermia group, arterial blood gases were analyzed at 37°C_BL_, and finally, after 5 h, also venous blood gases were analyzed. Arterial and venous blood oxygen content (CaO_2_ and CvO_2_), DO_2_, and oxygen consumption (V_O2_) were calculated using the following formulas: CaO_2_ and CvO_2_ = (Hb × 1.34 × SaO_2_/100) + (pO_2_ × 0.0031 × 7.5), D_O2_ = CaO_2_ × CO and V_O2_ = CO × (CaO_2_ – CvO_2_).

### Levosimendan and Nitroprusside

Levosimendan was purchased from Orion pharma as SIMDAX® (2.5 mg/ml). On the day of the experiment, it was diluted in 5% glucose (10). Dilution was calculated in each experiment to adjust for body weight.

Nitroprusside was purchased from Hospira as NITROPRESS® (25 mg/ml). On the day of the experiment, it was diluted to 0.125 mg/ml (1:200) in 5% glucose (8).

### Statistical Analyses

All statistical analysis were performed using SigmaPlot 13.0 (SAS).

The sample size was calculated with three independent sample size analysis. The first two used the expected difference between myocardial blood flow and mean arterial pressure after rewarming in the three group thermic groups. The third used the expected change in myocardial blood flow after rewarming compared to baseline.

Based on these sample size analyses, we concluded that a total of 27 animals were needed to attain a statistical power >0.8.

Hemodynamic results were analyzed with a two-way repeated measure ANOVA analysis. *Post hoc* analysis and all group comparisons were performed using a Holm–Sidak method.

Within-group comparisons of organ blood flow and blood gas data were done using repeated measure one-way ANOVA. *Post hoc* analyses were performed using a Holm–Sidak method. Between-group analyses at baseline, during, and after rewarming were done using a one-way ANOVA. *Post hoc* analyses were performed using a Holm–Sidak method.

## Results

### Organ Blood Flow

**Figure 1:**
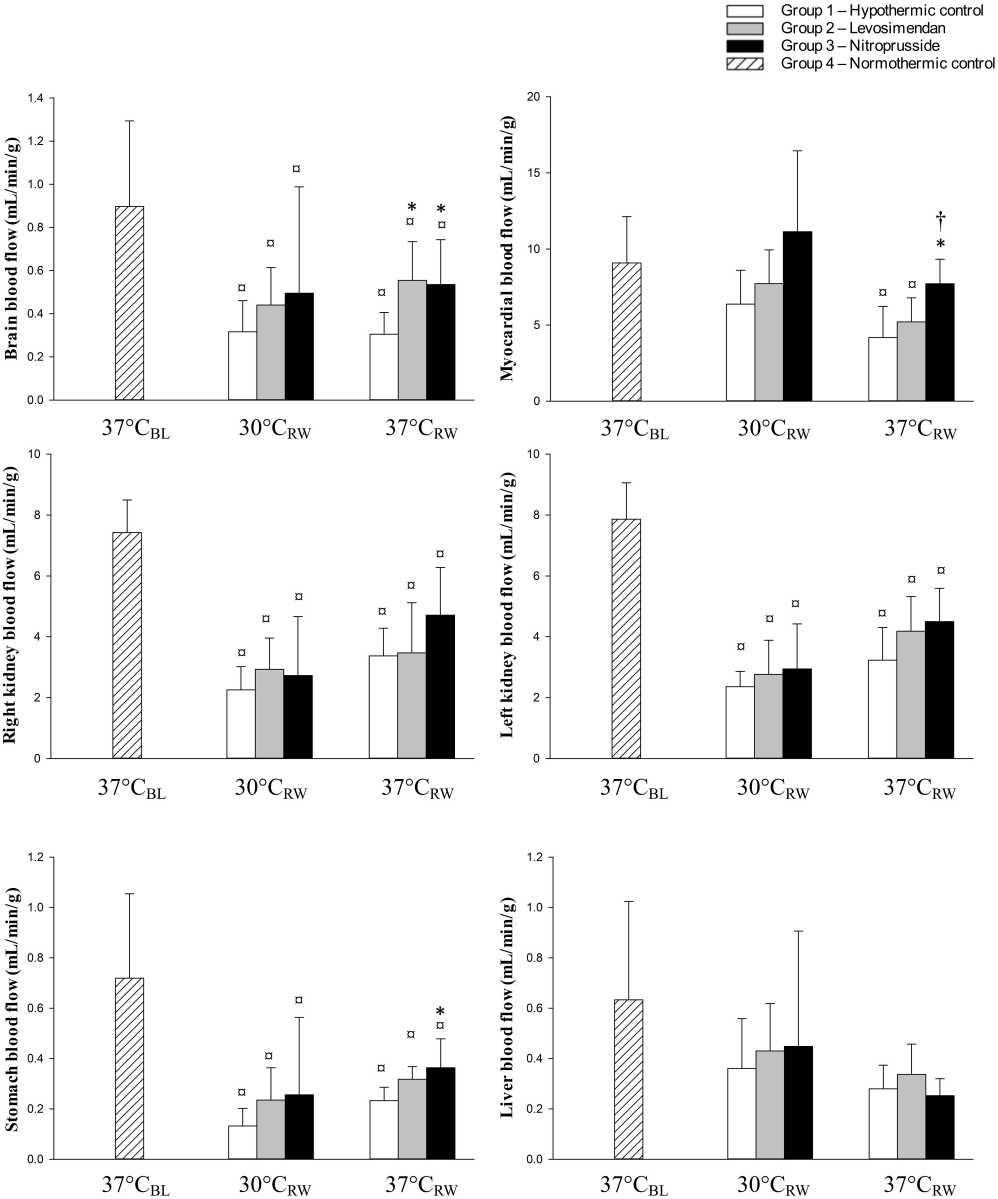
Organ blood flow at baseline (37°C_BL_), during and after rewarming (30 and 37°C_RW_) from hypothermia in untreated and treated rats with levosimendan or nitroprusside (each group *n* = 7). Values are mean ± SD. *P < 0.05 vs. control group at corresponding temperatures, ^†^*P* < 0.05 vs. levosimendan group at corresponding temperatures, ^¤^*P* < 0.05 vs. 37°C_BL_ (Holm–Sidak method).

Figure 1 compared to 37°C_BL_, blood flow in the brain, stomach, right, and left kidney was reduced in all groups at 30°C_RW_. No difference in blood flow of other organs was found between the three groups.

At 37°C_RW_, the control and LS-treated groups demonstrated significant reductions in blood flow to all organs, except for the liver, compared to 37°C_BL_ Similar results were found in the SNP group. However, heart blood flow was not reduced at 37°C_RW_

At 37°C_RW_, both LS and SNP elevated brain blood flow compared to the control group. Blood flow in the heart was elevated in the SNP-treated group compared to both the control group and LS. Blood flow in the stomach was elevated in the SNP group compared to the control group. No difference was found between the three groups in kidneys or liver blood flow.

### Hemodynamic Results

**Figure 2:**
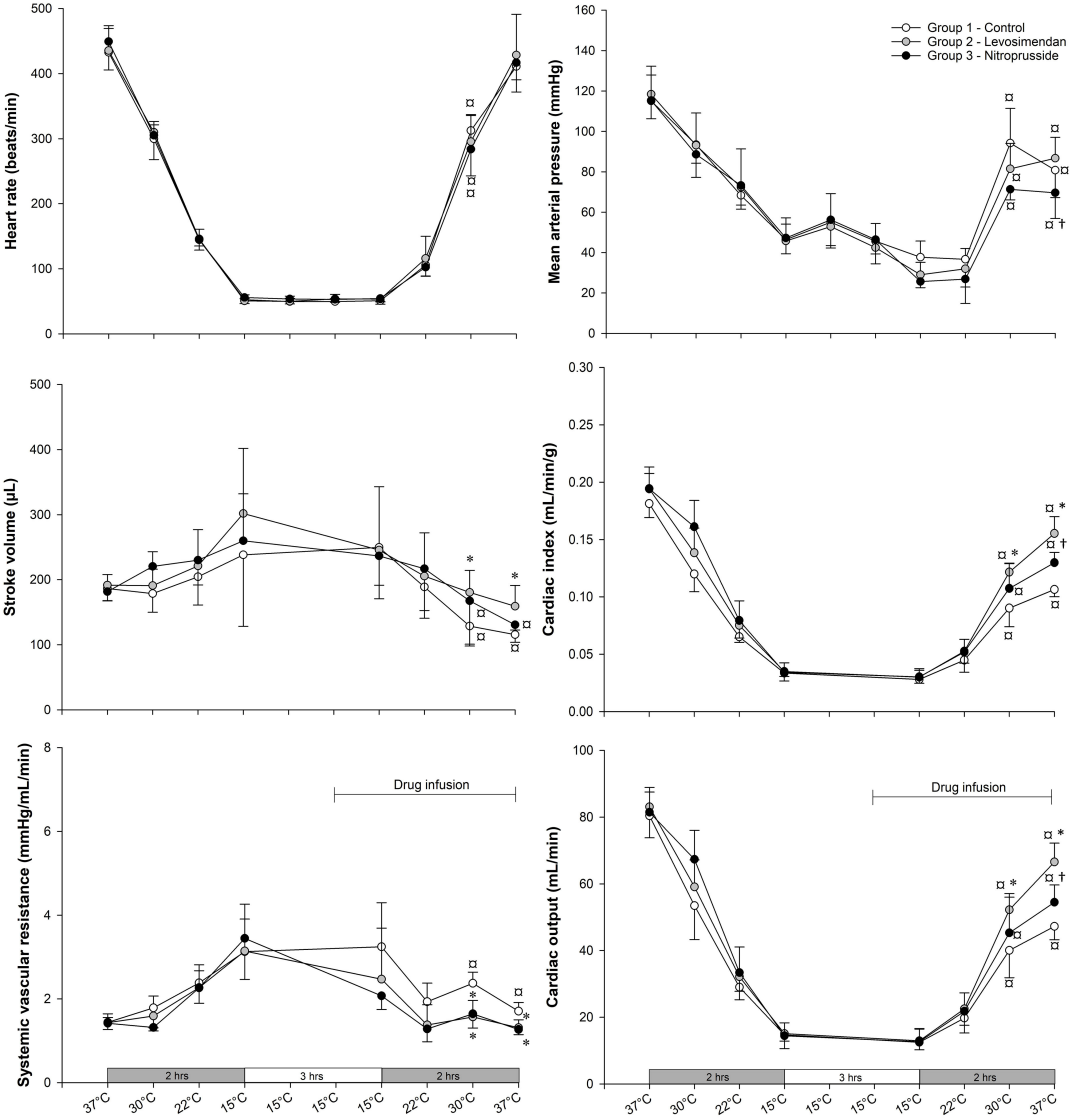
Heart rate (HR), mean arterial pressure (MAP), stroke volume (SV), cardiac output (CO), cardiac index (CI), and systemic vascular resistance (SVR) at baseline, during and after rewarming from hypothermia in untreated and treated rats with levosimendan or nitroprusside (each group *n* = 7). Values are mean ± SD. *P < 0.05 vs. control group at 37°C_BL_, 30°C_RW_, and 37°C_RW_; ^†^*P* < 0.05 vs. levosimendan group at corresponding temperatures; ^¤^*P* < 0.05 vs. 37°C_BL_ within-group (Holm–Sidak method).

Table 1 and Figure 2 no differences were found in any of the hemodynamic variables between the three hypothermia groups at baseline (37°C_BL_).

**Table 1:** Heart rate (HR), mean arterial pressure (MAP), stroke volume (SV), cardiac output (CO), cardiac index (CI), and systemic vascular resistance (SVR) at baseline (37°C_BL_), during and after rewarming (30 and 37°C_RW_) from hypothermia in untreated and treated rats with levosimendan or nitroprusside (each group *n* = 7).

**Parameter**	**Group**	**37^**°**^C_**BL**_**	**30^**°**^C_**RW**_**	**37$37_{\text{RW}}^{{}^{\circ}}$**
HR (beats/min)	Control	433 ± 27	312 ± 23^¤^	411 ± 39
	Levosimendan	435 ± 34	296 ± 41^¤^	428 ± 63
	Nitroprusside	449 ± 24	284 ± 41^¤^	417 ± 26
MAP (mmHg)	Control	115.1 ± 8.8	94.2 ± 17.1^¤^	80.8 ± 13.5^¤^
	Levosimendan	118.4 ± 13.8	81.5 ± 12.5^¤^	86.7 ± 10.3^¤^
	Nitroprusside	115.3 ± 12.6	71.3 ± 5.2^¤^^*^	69.6 ± 12.7^¤^^†^
SV (μL)	Control	186 ± 19	129 ± 27^¤^	116 ± 11^¤^
	Levosimendan	192 ± 16	180 ± 33^*^	159 ± 31^*^
	Nitroprusside	182 ± 13	167 ± 69^¤^	131 ± 8^¤^
CO (mL/min)	Control	80.41 ± 6.5	40.03 ± 8.1^¤^	47.22 ± 3.9^¤^
	Levosimendan	83.09 ± 5.8	52.24 ± 4.8^¤^^*^	66.57 ± 5.6^¤^^*^
	Nitroprusside	81.47 ± 6.1	45.28 ± 10.7^¤^	54.48 ± 5.2^¤^^†^
CI (mL/min/g)	Control	0.18 ± 0.02	0.09 ± 0.01^¤^	0.10 ± 0.01^¤^
	Levosimendan	0.19 ± 0.02	0.12 ± 0.01^¤^^*^^†^	0.15 ± 0.01^¤^^*^
	Nitroprusside	0.19 ± 0.01	0.10 ± 0.02^¤^	0.12 ± 0.01^¤^^*^
SVR (mmHg/mL/min)	Control	1.44 ± 0.17	2.37 ± 0.26^¤^	1.71 ± 0.20 ^¤^
	Levosimendan	1.43 ± 0.20	1.57 ± 0.26^*^	1.31 ± 0.18^*^
	Nitroprusside	1.42 ± 0.14	1.64 ± 0.31^*^	1.27 ± 0.12^*^

*Values are mean ± SD*.

* *P < 0.05 vs. control group at corresponding temperatures*.

† *P < 0.05 vs. levosimendan group at corresponding temperatures*.

¤ *P < 0.05 vs. 37°C_BL_ within-group (Holm–Sidak method)*.

At 30°C during rewarming (30°C_RW_), all three groups, independent of intervention, showed depressed MAP, CI, CO, and HR when compared to 37°C_BL_.

At 30°C_RW_, CI, CO, and SV were significantly higher in the LS group compared to the control. Further, SVR was reduced in both SNP and LS groups compared to the control. No differences were found between SNP and LS in any of the other hemodynamic variables.

Compared to 37°C_B_, SV was depressed in both the control and the SNP group at 37°C after rewarming (37°C_RW_). SVR was elevated only in the control group at 37°C_RW_ compared to 37°C_BL_. At 37°C_RW_, MAP, CI, and CO were all depressed in all three groups, while SV was depressed only in the SNP and control groups compared to 37°C_BL_.

At 37°C_RW_, CO, CI, and SV were significantly higher and SVR significantly lower in the LS-treated group compared to the control. Compared to the control group, SNP significantly elevated CO and CI and reduced MAP and SVR. CO, CI, and MAP were significantly higher with LS than SNP.

### Blood Gas Results

Table 2 no differences were found in arterial blood gases between the three hypothermia groups at 37°C_BL_.

**Table 2:** Hemoglobin (Hb), hematocrit, oxygen saturation (SaO_2_), oxygen saturation (pO_2_), arterial oxygen content (CaO_2_), venous oxygen content (CvO_2_), oxygen delivery, (*D*_*O*2_); oxygen consumption, (*V*_*O*2_), at baseline (37°C_BL_), during and after rewarming (30°C_RW_, and 37°C_RW_) from hypothermia in untreated and treated rats with levosimendan or nitroprusside (each group *n* = 7).

**Parameter**	**Group**	**37^**°**^C_**BL**_**	**30^**°**^C_**RW**_**	**37^**°**^C_**RW**_**
Hb (g/dL)	Control	12.56 ± 0.96	14.69 ± 0.25^¤^	13.27 ± 0.84^¤^
	Levosimendan	12.86 ± 1.18	14.29 ± 0.36^¤^	13.01 ± 0.88^¤^
	Nitroprusside	12.13 ± 0.71	14.04 ± 0.32^¤^	12.77 ± 0.62^¤^
Hct (%)	Control	38.63 ± 2.93	45.04 ± 0.74^¤^	40.80 ± 2.55^¤^
	Levosimendan	39.59 ± 3.53	43.80 ± 1.10^¤^	39.99 ± 2.73^¤^
	Nitroprusside	37.37 ± 2.12	43.13 ± 0.97^¤^	39.29 ± 1.85^¤^
SaO_2_ (%)	Control	86.29 ± 8.93	95.10 ± 0.77^¤^	91.80 ± 4.17^¤^
	Levosimendan	88.46 ± 3.46	91.66 ± 2.53	92.13 ± 3.71
	Nitroprusside	91.67 ± 2.98	94.76 ± 0.55	91.17 ± 4.28
pO_2_ (kPa)	Control	9.77 ± 2.39	17.38 ± 1.41	12.22 ± 2.54^¤^
	Levosimendan	9.55 ± 1.01	15.28 ± 2.38	11.12 ± 1.87^¤^
	Nitroprusside	12.16 ± 0.70	17.61 ± 0.85	12.31 ± 0.73^¤^
CaO_2_ (mg/dL)	Control	14.71 ± 1.61	18.98 ± 0.39	16.58 ± 0.74
	Levosimendan	15.49 ± 1.89	17.92 ± 0.79	16.32 ± 1.35
	Nitroprusside	15.16 ± 0.70	18.25 ± 0.48	15.88 ± 1.03
D_O2_ (mg/min)	Control	1,180 ± 142	766 ± 54^¤^	784.5 ± 89^¤^
	Levosimendan	1,281 ± 117	935 ± 51^¤^^*^	1083 ± 81^¤^^*^
	Nitroprusside	1,235 ± 103	820 ± 68^¤^	866 ± 111^¤^^†^
V_O2_ (mg/min)	Control	–	–	465 ± 40
	Levosimendan	–	–	586 ± 109^*^
	Nitroprusside	–	–	521 ± 110 ^†^
pH	Control	7.35 ± 0.03	7.22 ± 0.03^¤^	7.30 ± 0.04^¤^
	Levosimendan	7.35 ± 0.04	7.22 ± 0.01^¤^	7.33 ± 0.03^¤^
	Nitroprusside	7.38 ± 0.02	7.20 ± 0.02^¤^	7.28 ± 0.03^¤^
pCO_2_ (kPa)	Control	5.01 ± 0.88	5.01 ± 0.42	4.11 ± 0.56^¤^
	Levosimendan	5.07 ± 0.26	5.50 ± 0.22	4.31 ± 0.38^¤^
	Nitroprusside	4.52 ± 0.49	5.50 ± 0.17^¤^	4.63 ± 0.34
Lactate (mmol/L)	Control	0.90 ± 0.51	2.97 ± 0.23^¤^	3.64 ± 0.84^¤^
	Levosimendan	0.87 ± 0.51	3.01 ± 0.41^¤^	3.26 ± 0.67^¤^
	Nitroprusside	0.63 ± 0.19	3.93 ± 0.31^¤^^†^^*^	4.10 ± 0.52^¤^
BE (mmol)	Control	−4.60 ± 2.31	−11.80 ± 0.57^¤^	−10.16 ± 1.96^¤^
	Levosimendan	−3.94 ± 2.22	−10.23 ± 0.68^¤^	−8.41 ± 1.12^¤^
	Nitroprusside	−4.93 ± 1.45	−11.11 ± 0.67^¤^	−9.57 ± 1.07^¤^
${\text{HCO}}_{3}^{-}$ (mmol/L)	Control	20.44 ± 1.52	15.00 ± 0.58^¤^	16.54 ± 1.46^¤^
	Levosimendan	21.02 ± 1.87	15.82 ± 0.46^¤^	17.80 ± 0.90^¤^
	Nitroprusside	20.53 ± 1.01	15.25 ± 0.53^¤^	16.72 ± 0.84^¤^

*Values are mean ± SD*.

* *P < 0.05 vs. control group at corresponding temperatures*.

† *P < 0.05 vs. levosimendan group at corresponding temperatures*.

¤ *P < 0.05 vs. 37°C_BL_ within-group (Holm–Sidak method)*.

Compared to 37°C_BL_, Hb), hematocrit and lactate were significantly elevated at 30°C_RW_ in all groups. SaO_2_ was elevated in the control group, and pCO_2_ was elevated in the SNP group. Further, D_O2_, pH, base excess, and ${\text{HCO}}_{3}^{-}$ were significantly reduced in all groups at 30°C_RW_. In addition, SaO_2_ was decreased in the control group.

At 30°C_RW_, D_O2_ was significantly higher in the LS group than in the control. Further, plasma lactate levels were elevated in the SNP group compared to both control and LS groups. No other between-group differences were found at 30°C_RW_.

At 37°C_RW_ Hb, hematocrit, pO_2_, and lactate were elevated in all groups compared to 37°C_BL_. Further, D_O2_, pH, base excess, and ${\text{HCO}}_{3}^{-}$ were significantly reduced in all groups at 37°C_RW_. In addition, SaO_2_ was reduced in the control group.

At 37°C_RW_, D_O2_, and V_O2_ were significantly elevated in the LS-treated group than in the SNP and control. No differences were found between groups in other variables at 37°C_RW_.

## Discussion

The main findings of this study are that LS and SNP equally improved blood flow to the brain, despite elevating cardiac output to different levels. When comparing the effects of two different pharmacologic interventions during rewarming, vasodilation by SNP vs. inotropic support plus vasodilation by LS, we find that, although LS is superior to SNP to restore global hemodynamic function, OBF is equally or better preserved after intervention with SNP. This finding indicates that increased vascular resistance is a central element in the complex pathophysiology of cardiac dysfunction and reduced OBF after rewarming from hypothermia.

The aim of this experiment was to verify if the documented effects that both LS and SNP to elevate OBF during normothermic conditions (18–20) are valid also during rewarming from hypothermia. As a surrogate for monitoring organ microcirculatory variables, in clinical practice, we usually pay attention to variables such as CO, HR, MAP, and SVR. The specific aim was to test our hypotheses that the combined inotropic and vasodilatory effects of LS would improve OBF over that of the isolated vasodilation offered by SNP after rewarming from hypothermia. However, this experiment indicates that, during rewarming, peripheral vasodilation is superior to CO to increase OBF. To emphasize this, we present and discuss our data related to the different organs.

### Brain Blood Flow

A mismatch between brain blood flow (BBF) and cerebral metabolic rate of oxygen during hypothermia is a much-discussed topic (2, 26–29). Other studies state that, while the cerebral metabolic rate of oxygen is normalized, BBF remains reduced after rewarming. This indicates the existence of maintained dysfunctional cerebral autoregulation (26, 30, 31). Evidence of faulty cerebral autoregulation after rewarming is reported and supports the presence of a concomitant change in cerebral vascular function and hemodynamics (32–35). Under non-pathological, normothermic conditions, neither SNP nor LS will affect BBF (18, 36). In the present experiment, a 42% reduction in CO corresponds to a 75% reduction of BBF in the control group after rewarming. For comparison, studies during normothermic conditions on healthy humans report that a 30% reduction in CO would reduce BBF by only 10% (37). If we return to the present experiment, different from the non-treated control group, both SNP and LS elevated BBF similarly, 75 vs. 81%. However, at the same time, SNP managed to elevated CO by only 14%, compared to 30% with LS. Thus, we interpret our findings to disclose alterations of cerebral autoregulation after rewarming, possibly due to elevated cerebral vascular resistance (32, 33). To speculate, we suggest that differences in the effects of SNP and LS to elevate BBF over those to increase CO are due to increased cerebral vascular resistance. This increased vascular resistance appears not to be expedient, and the vasodilator properties of SNP and LS are the primary driving forces to improved BBF in this study.

### Myocardial Blood Flow

Aortic pressure and coronary resistance strongly regulate myocardial blood flow (MBF) by myocardial metabolic demand (38). While MBF is depressed after hypothermia, the autoregulatory properties of the coronaries appear to be unaltered. In this study, only the vasodilatory effects of SNP caused the elevation of MBF. This is in concordance with findings reported during normothermic conditions (18, 39).

Investigators have demonstrated a reduced MBF during hypothermia with spontaneous circulation (2, 40–43). Further, Berne revealed that the coronary regulation of flow is changed in hypothermia. He argued that the coronary vessels are relatively vasodilated during hypothermia, as the relative reduction in MBF is lower than the change in aortic perfusion pressure (41, 44). He stated that the effects of hypothermia on coronary smooth muscle are relaxation and that this is the main explanation for vasodilation, which causes a high ratio between MBF and myocardial oxygen consumption (41, 44).

In concordance with previous studies, the present study also shows a reduced MBF during and after rewarming (2, 40–43). Previously, we reported that the coronary vasculature has reduced sensitivity to endothelium-dependent and *independent* vasodilation but normalized after rewarming (45). This might indicate functioning vascular regulation after rewarming. Although this study made no attempts to investigate endothelium-dependent vasodilation in this study, we show that SNP-induced endothelium-independent vasodilation resulted in elevated MBF after rewarming from hypothermia.

### Myocardial Function

A study from our group demonstrated LS to improve cardiac contractility and CO after rewarming from hypothermia (10) after using the present intact animal model. In the present study, LS improved cardiac function without elevating MBF, as demonstrated using the same model and dosage of LS. Elevated cardiac contractility should elevate myocardial oxygen consumption (46). Our findings might indicate that MBF matches cardiac metabolic demands and that regulation of MBF is preserved after rewarming. A similar conclusion was made in earlier studies (25, 47). Lastly, in another study, LS did not increase plasma cardiac troponin I, compared to non-treated animals during rewarming. As the release of cardiac troponin is a marker of myocardial damage, we understand this to indicate the absence of further damaging factors such as hypoxia or apoptosis (10).

### Renal and Stomach Blood Flow

Previous studies have reported depressed renal blood flow (RBF) following rewarming (2, 48). As both LS and SNP failed to elevate RBF in response to elevated CO, the depressed flow likely stems from other mechanisms than low CO. Hypothermia and rewarming is associated with activation of the renin–aldosterone–angiotensin system (RAAS) in both humans and rats (49, 50). Broome et al. demonstrated that SNP did not affect RBF during targeted vasoconstriction with angiotensin II infusion (19). They interpreted that the vasoconstrictive effects of angiotensin II supersede the vasodilatory effects of SNP in the kidney. In the presented study, SNP failed to elevate RBF. As in the experiment with Broome et al., the elevation of the renin–aldosterone–angiotensin system might explain our findings (49, 50).

In contrast to the RBF, Broome et al. found SNP to have a vasodilating effect on stomach blood flow (SBF) during angiotensin II-induced vasoconstriction (19). In the presented study, SNP also elevated SBF. To speculate, our findings might indicate that activation of the renin–aldosterone–angiotensin system hormones as possible mediators of poor RBF and SBF. Our results and reports from other investigators may support these ideas, as they show that SNP only affects SBF and not RBF, as well as increased renin–aldosterone–angiotensin during hypothermia (49, 50). Further, studies from an identical model, as presented, show renal tubular necrosis after rewarming (51). Severe tubular necrosis is associated with renal vasoconstriction and reduced RBF and explains why SNP and LS failed to elevate RBF.

### Clinical Significance

The physiological message this study brings to the clinical table is to focus on the mismatch between organ perfusion/microcirculation and global perfusion in the hypothermia/rewarming setting. Routine bedside intensive care lacks tools to assess organ perfusion and microcirculation changes, continually and accurately, in response to treatment. This highlights the need to be cautious in translating variables related to global circulation into changes that are important for organ blood flow and microcirculation, in this case, during rewarming from hypothermia.

This experiment, describing both LS and SNP's pharmacologic effects to alleviate post-hypothermic circulatory dysfunction and OBF in a rodent model, may encourage further research in large animal models before clinical applications can be suggested.

### Limitations

SNP is a known cyanide donor (52, 53). Enzymatic breakdown of cyanide is done by the enzyme thiosulfate sulfurtransferase (54). In critically ill patients, SNP-induced cyanide breakdown is higher than production at SNP infusion rates below 2 μg/kg/min (55). However, studies have reported elevated cyanide levels at lower infusion rates during hypothermia, possibly due to low enzymatic activity. Therefore, investigators have advocated for caution when using high doses or prolonged use of SNP (56). Animals in this study received, on average, 2.9 μg/kg/min SNP and are, therefore, possible victims of high cyanide levels, affecting the results. We do see possible evidence of cyanide in the presented data. Both elevated venous O_2_ and serum lactate are present in the SNP group, although not significantly. Other investigators have toned down the importance of cyanide poisoning by SNP, also during hypothermia and cardiopulmonary bypass. Interestingly enough, the SNP-treated animals, despite possible cyanide toxicity, had improved hemodynamic and blood flow parameters compared to the control group (52, 53).

## Conclusions

From critical care reports, we know that efforts to elevate OBF will improve end-organ function and patient survival. The present findings indicate potential beneficial effects on organ function by combining cardiac inotropic support and reducing peripheral organ vascular resistance (15–17). In more detail, our results demonstrate the beneficial effects of vasodilation to increase CO and OBF, in general, and BBF, in particular. While the inotropic effects of LS are shown to improve CO, its relative weak additional vasodilator properties fail to improve peripheral organ circulation. We, therefore, interpret the vasodilator properties of LS and SNP to be the primary source to increase organ blood flow, superior to the increase in CO.

## Data Availability Statement

The raw data supporting the conclusions of this article will be made available by the authors, without undue reservation.

## Ethics Statement

The animal study was reviewed and approved by Norwegian Animal Research Authority.

## Author Contributions

BH has contributed with research ideas, protocol development, experimental work, data analysis, and manuscript. TK has contributed with experimental work and data analysis. ED has contributed with research ideas, data analysis, and manuscript. TT has contributed with research ideas, protocol development, data analysis, and manuscript. All authors contributed to the article and approved the submitted version.

## Conflict of Interest

The authors declare that the research was conducted in the absence of any commercial or financial relationships that could be construed as a potential conflict of interest.

## Abbreviations

SVR: systemic vascular resistance
LS: levosimendan
SNP: sodium nitroprusside
CO: cardiac output
OBF: organ blood flow
DO_2_: oxygen transport
HR: heart rate
SV: stroke volume
CI: cardiac index
SaO_2_: oxygen saturation
Hb: hemoglobin
CaO_2_: Arterial blood oxygen content
CvO_2_: venous blood oxygen content
VO_2_: oxygen consumption
MAP: mean arterial pressure
BBF: brain blood flow
MBF: myocardial blood flow
RBF: renal blood flow
SBF: stomach blood flow.

## References

[1] Danzl DF Pozos RS. Accidental hypothermia. N Engl J Med. (1994) 331:1756–60. 10.1056/NEJM199412293312607 7984198

[2] Tveita T Ytrehus K Skandfer M Oian P Helset E Myhre ES. Changes in blood flow distribution and capillary function after deep hypothermia in rat. Can J Physiol Pharmacol. (1996) 74:376–81. 10.1139/y96-028 8828884

[3] Tveita T Mortensen E Hevroy O Refsum H Ytrehus K. Experimental hypothermia: effects of core cooling and rewarming on hemodynamics, coronary blood flow, and myocardial metabolism in dogs. Anesth Analg. (1994) 79:212–8. 10.1213/00000539-199408000-00002 7639353

[4] Han Y-S Tveita T Kondratiev TV Prakash YS Sieck GC. Changes in cardiovascular beta-adrenoceptor responses during hypothermia. Cryobiology. (2008) 57:246–50. 10.1016/j.cryobiol.2008.09.006 18834873

[5] Tveita T Ytrehus K Myhre ES Hevroy O. Left ventricular dysfunction following rewarming from experimental hypothermia. J Appl Physiol. (1998) 85:2135–9. 10.1152/jappl.1998.85.6.2135 9843536

[6] van der Ploeg G-J Goslings JC Walpoth BH Bierens JJLM. Accidental hypothermia: rewarming treatments, complications and outcomes from one university medical centre. Resuscitation. (2010) 81:1550–5. 10.1016/j.resuscitation.2010.05.023 20702016

[7] Mégarbane B Axler O Chary I Pompier R. Hypothermia with indoor occurrence is associated with a worse outcome. J Intensive Care Med. (2000) 26:1843–9. 10.1007/s001340000702 11271094

[8] Håheim B Kondratiev T Dietrichs ES Tveita T. The beneficial hemodynamic effects of afterload reduction by sodium nitroprusside during rewarming from experimental hypothermia. Cryobiology. (2017) 77:75–81. 10.1016/j.cryobiol.2017.05.002 28479295

[9] Soar J Perkins GD Abbas G Alfonzo A Barelli A Bierens JJLM. European resuscitation council guidelines for resuscitation 2010 section 8. Cardiac arrest in special circumstances: Electrolyte abnormalities, poisoning, drowning, accidental hypothermia, hyperthermia, asthma, anaphylaxis, cardiac surgery, trauma, pregnancy, electrocution. Resuscitation. (2010) 81:1400–33. 10.1016/j.resuscitation.2010.08.015 20956045

[10] Dietrichs ES Håheim B Kondratiev T Sieck GC Tveita T. Cardiovascular effects of levosimendan during rewarming from hypothermia in rat. Cryobiology. (2014) 69:402–10. 10.1016/j.cryobiol.2014.09.007 25280932

[11] Dietrichs ES Kondratiev T Tveita T. Milrinone ameliorates cardiac mechanical dysfunction after hypothermia in an intact rat model. Cryobiology. (2014) 69:361–6. 10.1016/j.cryobiol.2014.09.002 25224046

[12] Pathak A Lebrin M Vaccaro A Senard JM Despas F. Pharmacology of levosimendan: inotropic, vasodilatory and cardioprotective effects. J Clin Pharm Ther. (2013) 38:341–9. 10.1111/jcpt.12067 23594161

[13] Mohan P Brutsaert DL Paulus WJ Sys SU. Myocardial contractile response to nitric oxide and cGMP. Circulation. (1996) 93:1223–9. 10.1161/01.CIR.93.6.1223 8653845

[14] Thompson RB Bos EJ Esposito DJ. The effects of acute afterload change on systolic ventricular function in conscious dogs with normal vs. failing hearts. Eur J Heart Fail. (2003) 5:741–9. 10.1016/S1388-9842(03)00152-1 14675852

[15] Spronk PE Zandstra DF Ince C. Bench-to-bedside review: sepsis is a disease of the microcirculation. Crit Care. (2004) 8:462–8. 10.1186/cc2894 15566617 PMC1065042

[16] Trzeciak S McCoy JV Phillip Dellinger R Arnold RC Rizzuto M Abate NL. Early increases in microcirculatory perfusion during protocol-directed resuscitation are associated with reduced multi-organ failure at 24 h in patients with sepsis. Intensive Care Med. (2008) 34:2210–7. 10.1007/s00134-008-1193-6 18594793 PMC2821162

[17] Tuchschmidt J Fried J Astiz M Rackow E. Elevation of cardiac output and oxygen delivery improves outcome in septic shock^*^. Chest. (2006) 102:216–20. 10.1378/chest.102.1.216 1623756

[18] Pagel PS Hettrick DA Warltier DC. Influence of levosimendan, pimobendan, and milrinone on the regional distribution of cardiac output in anaesthetized dogs. Br J Pharmacol. (1996) 119:609–15. 10.1111/j.1476-5381.1996.tb15716.x 8894186 PMC1915696

[19] Broomé M Åneman A Haney M HÄggmark S Johansson G Biber B. Angiotensin II mesenteric and renal vasoregulation: dissimilar modulatory effects with nitroprusside. Acta Anaesthesiol Scand. (2000) 44:1238–45. 10.1034/j.1399-6576.2000.441009.x 11065204

[20] Rowe GG Henderson RH. Systemic and coronary hemodynamic effects of sodium nitroprusside. Am Heart J. (1974) 87:83–7. 10.1016/0002-8703(74)90394-9 4808762

[21] Kondratiev TV Myhre ESP Simonsen O Nymark TB Tveita T. Cardiovascular effects of epinephrine during rewarming from hypothermia in an intact animal model. J Appl Physiol. (2006) 100:457–64. 10.1152/japplphysiol.00356.2005 16210439

[22] Reinhardt CP Dalhberg S Tries MA Marcel R Leppo JA. Stable labeled microspheres to measure perfusion: validation of a neutron activation assay technique. Am J Physiol Heart Circ Physiol. (2001) 280:H108–16. 10.1152/ajpheart.2001.280.1.H108 11123224

[23] Ishise S Pegram BL Yamamoto J Kitamura Y Frohlich ED. Reference sample microsphere method: cardiac output and blood flows in conscious rat. Am J Physiol. (1980) 239:443–9. 10.1152/ajpheart.1980.239.4.H443 7425136

[24] Valkov S Mohyuddin R Nilsen JH Schanche T Kondratiev TV Sieck GC. Organ blood flow and O2 transport during hypothermia (27°C) and rewarming in a pig model. Exp Physiol. (2019) 104:50–60. 10.1113/EP087205 30375081

[25] Kondratiev TV Flemming K Myhre ESP Sovershaev MA Tveita T. Is oxygen supply a limiting factor for survival during rewarming from profound hypothermia? Am J Physiol Heart Circ Physiol. (2006) 291:H441–50. 10.1152/ajpheart.01229.2005 16461371

[26] Michenfelder JD Milde JH. The effect of profound levels of hypothermia (below 14 C) on canine cerebral metabolism. J Cereb Blood Flow Metab. (1992) 12:877–80. 10.1038/jcbfm.1992.120 1506453

[27] Yenari MA Wijman CAG Steinberg GK Effects of Hypothermia on Cerebral Metabolism, Blood Flow, and Autoregulation. In: Mayer SA, Sessler DI, editors. Therapeutic Hypothermia (Marcel Dekker) (2005). p. 141–79.

[28] Polderman KH. Application of therapeutic hypothermia in the ICU: opportunities and pitfalls of a promising treatment modality. Part 1: indications evidence. Intens Care Med. (2004) 30:556–75. 10.1007/s00134-003-2152-x 14767591

[29] Polderman KH Application of therapeutic hypothermia in the intensive care unit. Intensive Care Med. (2004) 30:757–69. 10.1007/s00134-003-2151-y 14767590

[30] Michenfelder JD Milde JH. The relationship among canine brain temperature, metabolism, and function during hypothermia. Anesthesiology. (1991) 75:130–6. 10.1097/00000542-199107000-00021 2064037

[31] Murkin JM Farrar JK Tweed WA McKenzie FN Guiraudon G. Cerebral autoregulation and flow/metabolism coupling during cardiopulmonary bypass: the influence of PaCO2. Anesth Analg. (1987) 66:825–32. 10.1213/00000539-198709000-00003 3113288

[32] Johnston WE Vinten-Johansen J DeWitt DS O'Steen WK Stump DA Prough DS. Cerebral perfusion during canine hypothermic cardiopulmonary bypass: effect of arterial carbon dioxide tension. Ann Thorac Surg. (1991) 52:479–89. 10.1016/0003-4975(91)90909-A 1910323

[33] Mezrow CK Sadeghi AM Gandsas A Shiang HH Levy D Green R. Cerebral blood flow and metabolism in hypothermic circulatory arrest. Ann Thorac Surg. (1992) 54:609–16. 10.1016/0003-4975(92)91002-Q 1417216

[34] Hansen TN Dawson PE Brockbank K. Effects of hypothermia upon endothelial cells: mechanisms and clinical importance. Cryobiology. (1994) 31:101–6. 10.1006/cryo.1994.1013 8156796

[35] Wagerle LC Russo P Dahdah NS Kapadia N Davis DA. Endothelial dysfunction in cerebral microcirculation during hypothermic cardiopulmonary bypass in newborn lambs. J Thorac Cardiovasc Surg. (1998) 115:1047–54. 10.1016/S0022-5223(98)70404-0 9605074

[36] Crockard HA Brown FD Mullan JF. Effects of trimethaphan and sodium nitroprusside on cerebral blood-flow in rhesus-monkeys. Acta Neurochir. (1976) 35:85–9. 10.1007/BF01405936 822698

[37] Lassen NA Cerebral blood flow and oxygen consumption in man. Physiol Rev. (1959) 39:183–238. 10.1152/physrev.1959.39.2.183 13645234

[38] Rubio R Berne RM. Regulation of coronary blood flow. Prog Cardiovasc Dis. (1975) 18:105–22. 10.1016/0033-0620(75)90001-8 1099615

[39] Hoffman WE Satinover I Miletich DJ Albrecht RF Gans BJ Cardiovascular changes during sodium nitroprusside or adenosine triphosphate infusion in the rat. Anesth Analg. (1982) 61:99–103. 10.1213/00000539-198202000-00006 7198884

[40] Anzai T Turner MD Gibson WH Neely WA. Blood flow distribution in dogs during hypothermia and posthypothermia. Am J Physiol. (1978) 234:H706–10. 10.1152/ajpheart.1978.234.6.H706 665785

[41] Berne RM. Cardiodynamics and the coronary circulation in hypothermia. Ann N Y Acad Sci. (1959) 80:365–83. 10.1111/j.1749-6632.1959.tb49217.x 13799728

[42] Edwards WS Tuluy S Reber WE Siegel A Bing RJ. Coronary blood flow and myocardial metabolism in hypothermia. Ann Surg. (1954) 139:275. 10.1097/00000658-195403000-00003 13149071 PMC1609415

[43] Burlington RF Dean MS Jones SB. Coronary autoregulation and metabolism in hypothermic rat and ground squirrel hearts. Am J Physiol Regul Integr Comp Physiol. (1989) 256:R357–65. 10.1152/ajpregu.1989.256.2.R357 2916694

[44] Berne RM. The effect of immersion hypothermia on coronary blood flow. Circ Res. (1954) 2:236–42. 10.1161/01.RES.2.3.236 13161131

[45] Tveita T Hevroy O Refsum H Ytrehus K. Coronary endothelium-derived vasodilation during cooling and rewarming of the in situ heart. Can J Physiol Pharmacol. (1999) 77:56–63. 10.1139/y98-149 10535667

[46] Graham TP Covell JW Sonnenblick EH Ross J Braunwald E. Control of myocardial oxygen consumption: relative influence of contractile state and tension development. J Clin Invest. (1968) 47:375–85. 10.1172/JCI105734 12066781 PMC297180

[47] Tveita T Skandfer M Refsum H Ytrehus K. Experimental hypothermia and rewarming: changes in mechanical function and metabolism of rat hearts. J Appl Physiol. (1996) 80:291–7. 10.1152/jappl.1996.80.1.291 8847317

[48] Karim F Reza H. Effect of induced hypothermia and rewarming on renal hemodynamics in anesthetized dogs. Life Sci. (1970) 9:1153–63. 10.1016/0024-3205(70)90148-7 5481739

[49] Kuroda T Shida H Inokawak K Morimoto M Ikeda Y Tsugana J. Significance of renin-angiotensin system during and after surface-induced simple hypothermia in open-heart surgery. Jap Circ J. (1983) 47:400–5. 10.1253/jcj.47.400 6300482

[50] Munday KA Noble AR. Renin secretion in the hypothermic dog. J Physiol. (1970) 206:38–9P. 5498490

[51] Tveita T Johansen K Lien AH Myklebust R Lindal S. Morphologic changes in tubular cells from in situ kidneys following experimental hypothermia and rewarming. APMIS. (2005) 113:13–20. 10.1111/j.1600-0463.2005.apm1130103.x 15676010

[52] Undquist PL Rosling H Tydén H. Cyanide release from sodium nitroprusside during coronary bypass in hypothermia. Acta Anaesthesiol Scand. (1989) 33:686–8. 10.1111/j.1399-6576.1989.tb02992.x 2589001

[53] Thomas C Svehla L Moffett BS. Sodium-nitroprusside-induced cyanide toxicity in pediatric patients. Expert Opin Drug Saf. (2009) 8:599–602. 10.1517/14740330903081717 19645589

[54] Smith RP Kruszyna H. Nitroprusside produces cyanide poisoning via a reaction with hemoglobin. J Pharmacol Exp Ther. (1974) 191:557–63. 4427294

[55] Johanning RJ Zaske DE Tschida SJ Johnson SV Hoey LL Vance-Bryan K. A retrospective study of sodium nitroprusside use and assessment of the potential risk of cyanide poisoning. Pharmacotherapy. (1995) 15:773–7. 8602386

[56] Friederich JA Butterworth JF. Sodium nitroprusside: twenty years and counting. Anesth Analg. (1995) 81:152–62. 10.1097/00000539-199507000-00031 7598246

